# Projection of the future diabetes burden in the United States through 2060

**DOI:** 10.1186/s12963-018-0166-4

**Published:** 2018-06-15

**Authors:** Ji Lin, Theodore J. Thompson, Yiling J. Cheng, Xiaohui Zhuo, Ping Zhang, Edward Gregg, Deborah B. Rolka

**Affiliations:** 1 Centers for Disease Control and Prevention, Division of Diabetes Translation, Atlanta, USA; 2 Merck Research Laboratories, North Wales, USA

## Abstract

**Background:**

In the United States, diabetes has increased rapidly, exceeding prior predictions. Projections of the future diabetes burden need to reflect changes in incidence, mortality, and demographics. We applied the most recent data available to develop an updated projection through 2060.

**Methods:**

A dynamic Markov model was used to project prevalence of diagnosed diabetes among US adults by age, sex, and race (white, black, other). Incidence and current prevalence were from the National Health Interview Survey (NHIS) 1985–2014. Relative mortality was from NHIS 2000–2011 follow-up data linked to the National Death Index. Future population estimates including birth, death, and migration were from the 2014 Census projection.

**Results:**

The projected number and percent of adults with diagnosed diabetes would increase from 22.3 million (9.1%) in 2014 to 39.7 million (13.9%) in 2030, and to 60.6 million (17.9%) in 2060. The number of people with diabetes aged 65 years or older would increase from 9.2 million in 2014 to 21.0 million in 2030, and to 35.2 million in 2060. The percent prevalence would increase in all race-sex groups, with black women and men continuing to have the highest diabetes percent prevalence, and black women and women of other race having the largest relative increases.

**Conclusions:**

By 2060, the number of US adults with diagnosed diabetes is projected to nearly triple, and the percent prevalence double. Our estimates are essential to predict health services needs and plan public health programs aimed to reduce the future burden of diabetes.

**Electronic supplementary material:**

The online version of this article (10.1186/s12963-018-0166-4) contains supplementary material, which is available to authorized users.

## Background

The number of US adults aged 18 years or older with diagnosed diabetes quadrupled from 5.5 million in 1980 to 21.9 million in 2014, corresponding to a nearly three-fold increase in the percent prevalence from 3.5 to 9.1% [1]. Projections of the future diabetes burden are essential for predicting future needs for health care services, projecting future economic burden associated with the disease, and prioritizing public health programs to reduce the future burden of the disease.

Projections from previous studies have been lower than the observed prevalence. For example, King et al. (in 1998) [2] and Boyle et al. (in 2001) [3] predicted the diabetes population would reach 20 million and 9% of the population by 2025. Data from the National Health Interview Survey (NHIS) showed that those projected levels were already reached in 2010 [1]. The most recent projection (2006) predicted a prevalence of 6.8% for year 2010, which was more than two percentage points lower than estimates from survey data [4].

There are several possible reasons why projections from previous studies underestimated diabetes prevalence. For example, King et al. used a static model and assumed a constant diabetes prevalence. However, the prevalence of diabetes varies due to changes in incidence and the demographic composition of the population [2]. Later studies avoided this problem by using dynamic models. However, some key assumptions of those models (e.g., constant relative mortality risk across different age groups) were based on expert opinion and lacked empirical support [3–6]. Moreover, the data used in those studies do not reflect recent changes in the diabetes epidemic. Prevalence, incidence, and mortality have all changed since the previous projection studies. Diabetes incidence increased until 2008, but has stabilized at a slightly lower level since then [7]. Mortality rates for people both with and without diabetes have declined [8, 9]. The decline of mortality in the diabetes population means more people live with diabetes, and the decline in mortality in the non-diabetes population means more people are at risk of developing diabetes before they die.

In this study, we used a dynamic Markov model to project the number and percent of US adults with diagnosed diabetes through the year 2060. Our study advances the previous studies in three ways. First, since our model is dynamic, the future prevalence and population with diabetes changes according to annual changes in prevalence and demographics. Second, our model is driven by observed data with minimal additional assumptions about the parameters. Third, we used the latest data available to estimate the model parameters, so our projection is based on relatively stable estimates of diabetes incidence over the past 30 years, including the lower incidence rates observed since 2008.

## Methods

### The dynamic Markov model

We used a dynamic Markov model similar to the models used by Boyle et al. [3] and Honeycutt et al. [5]. The model contains three system states – No Diabetes, Diabetes, and Death – in which diabetes is defined as diagnosed diabetes. We divided the population by race (white, black, other) and sex (male, female) for a total of six sub-cohorts. Other race includes American Indians, Alaska Natives, Asians, Native Hawaiians, Pacific Islanders, and people identifying with two or more races. We combined these groups to have sufficient sample size for reliable estimates. In the model, each sub-cohort runs its own Markov chain to project the diabetes total population and percent prevalence each year through 2060. The system starts with an initial diabetes prevalence in each race-sex cohort and changes annually. A proportion of the No Diabetes population transitions to the Diabetes state each year according to transition probabilities governed by estimated diabetes incidence rates. Transition back to the No Diabetes state is not considered in the model due to the low probability of remission and high relapse rate [10]. Transitions to the Death state from the No Diabetes and Diabetes states are determined by the Census projection of death and the relative mortality risk of diabetes. The model is solved by Monte Carlo simulation; details are described in the Additional file 1: Appendix.

### Estimation of the model parameters

#### Initial diabetes prevalence

The 2014 diabetes prevalence for each race-sex cohort served as the starting point for the projection. Diabetes was defined as self-reported diagnosed diabetes. We used a logistic regression model to estimate single-year race-sex-specific prevalence using data from the 1985–2014 NHIS [11], a representative survey of the US non-institutionalized civilian population. The independent variables included in the final regression model were race, sex, age, squared and cubic terms of age, and all second order interaction terms between sex, age, and survey year. The 2014 prevalence was estimated by the single-year predictive margin for that year.

#### Diabetes incidence

We also used a logistic regression model to estimate diabetes incidence. Incidence was defined based on self-reported diagnosis during the prior year. The independent variables were the same as in the prevalence model except for the survey year variable. As the incidence rate was much smaller than the prevalence, we replaced the single year variable with periods (1985–1989, 1990–1994, 1995–1999, 2000–2005, and 2006–2014) to increase the sample size. As diabetes incidence rates had increased for 30 years and then plateaued since 2008 [7], we assumed future diabetes incidence rates would be similar to incidence rates during 2006–2014 in the base case simulation.

The prevalence and incidence models were estimated using SAS version 9.3 (SAS Institute Inc., Cary, NC, USA) with a maximum likelihood estimation method that accounted for the complex sample design.

#### Population and mortality

The demographic changes in the model were determined by the race-sex-specific number of people who became 18 years old, and the age-race-sex-specific numbers who migrated or died. All these parameters were obtained from the middle series of the US Census 2014 National Population Projection for the period 2014 to 2060.

The diabetes relative mortality risk was determined by the ratio of the mortality rates for persons with and without diabetes. The two sets of age-race-sex-specific rates were estimated by a Poisson regression model with discretized survival time using the NHIS 2000–2011 survey linked to the National Death Index (NDI). The independent variables included in the Poisson regression were race, sex, age, diabetes status, age squared, and interaction terms between age and all other independent variables. The detailed mortality model was described elsewhere [12]. The model was estimated using Stata version 13.1 (StataCorp LP, College Station, TX). The estimated diabetes relative mortality risk for years 2005–2011 was used in the Markov model to allocate Census projected deaths to the Diabetes and No Diabetes states.

### Model simulation

The Markov model simulation projects the future number and percent prevalence of diabetes in the US population. The model is represented by difference equations (see Additional file 1: Appendix) that include all the estimated transition probabilities from the regression models, and is solved using Monte Carlo simulation. We used the maximum-likelihood estimates from the regression models as the base case transition probabilities in the Markov model. Confidence intervals for the projection were generated by sampling 5000 times from the joint distribution of the regression model coefficients, and running the model to produce a projection series for each sample. R version 3.2.5 was used for the computation.

### Sensitivity analysis

Incidence rate is a critical parameter that affects the future projection. To assess the effect of higher or lower incidence rate scenarios, we varied the incidence by ±20% from the base case incidence. The higher incidence represents a worst case scenario in which diabetes incidence would rise due to increases in risk factors such as obesity. The lower incidence may represent the more optimistic scenario in which future diabetes incidence is reduced by widely implementing effective diabetes prevention strategies such as lifestyle interventions.

## Results

**Fig. 1:**
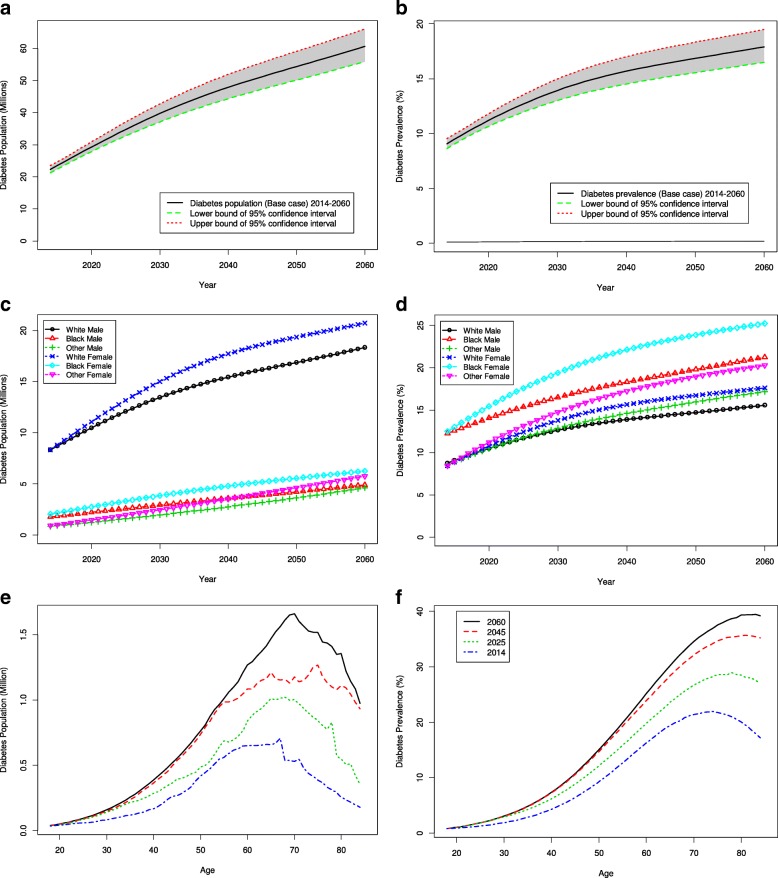
Projection of diagnosed diabetes prevalence in US adults: **a** overall number, **b** overall percent, **c** number by race and sex, **d** percent by race and sex, **e** number by age and year, **f** and percent by age and year

Both the number and percent prevalence of diagnosed diabetes among US adults are projected to increase continually through 2060 (Table 1 and Fig. 1a, b). The number (percent) of adults with diagnosed diabetes would increase from 22.3 million (9.1%) in 2014 to 39.7 million (13.9%) in 2030, and then reach 60.6 million (17.9%) in 2060.

**Table 1:** Projection of the future number (in millions) and percent (%) prevalence of US adults with diagnosed diabetes, by age group for selected years 2014–2060

Population (Millions)					
Age group (in years)					
	18–44	45–64	65–74	> = 75	Total
2014	2.86	10.27	5.51	3.67	22.31
	(2.67, 3.07)	(9.79, 10.77)	(5.28, 5.75)	(3.46, 3.89)	(21.19, 23.48)
2020	3.84	12.1	8.01	5.32	29.27
	(3.53, 4.21)	(11.54, 12.71)	(7.70, 8.34)	(5.07, 5.60)	(27.84, 30.86)
2030	5.01	13.67	10.92	10.11	39.71
	(4.45, 5.68)	(12.72, 14.71)	(10.34, 11.57)	(9.58, 10.73)	(37.09, 42.69)
2040	5.32	16.42	11.22	14.89	47.86
	(4.69, 6.08)	(15.11, 17.87)	(10.50, 12.00)	(14.00, 15.92)	(44.30, 51.87)
2050	5.51	18.94	12.86	16.99	54.3
	(4.85, 6.30)	(17.38, 20.66)	(11.98, 13.81)	(15.88, 18.27)	(50.09, 59.06)
2060	5.75	19.71	15.69	19.48	60.63
	(5.06, 6.58)	(18.07, 21.54)	(14.60, 16.88)	(18.14, 21.01)	(55.86, 66.01)
Prevalence (%)					
Age group (in years)					
	18–44	45–64	65–74	> = 75	Total
2014	2.5	12.3	20.8	18.5	9.1
	(2.3, 2.6)	(11.7, 12.9)	(19.9, 21.7)	(17.4, 19.6)	(8.6, 9.5)
2020	3.2	14.4	24.1	22.7	11.2
	(2.9, 3.5)	(13.7, 15.1)	(23.2, 25.1)	(21.7, 23.9)	(10.6, 11.8)
2030	3.9	16.5	27.7	28.6	13.9
	(3.5, 4.4)	(15.4, 17.8)	(26.2, 29.3)	(27.1, 30.3)	(13.0, 15.0)
2040	4.1	17.9	30.4	31.8	15.7
	(3.6, 4.7)	(16.5, 19.5)	(28.5, 32.5)	(29.9, 34.0)	(14.5, 17.0)
2050	4.1	19.1	32.3	33.9	16.8
	(3.6, 4.7)	(17.6, 20.9)	(30.1, 34.7)	(31.7, 36.4)	(15.5, 18.3)
2060	4.2	19.6	33.8	36	17.9
	(3.7, 4.8)	(17.9, 21.4)	(31.5, 36.4)	(33.5, 38.8)	(16.5, 19.5)

These projections represent the most likely (base case) scenario. 95% confidence intervals are given in parentheses

Over time, the overall diabetes percent prevalence is projected to increase by 0.3% per year, on average, before 2030, and by 0.1% per year after 2030 (Fig. 1b). The overall diabetes population size will increase by an average of 1.0 million people per year before 2030 and by 0.6 million per year thereafter (Fig. 1a).

The increase would vary by age group (Table 1 and Fig. 1e, f). People aged 65 years or older would have larger increases in both number and percent prevalence than younger adults. The number (percent) of people with diagnosed diabetes in the 65 years or older group would increase from 9.18 million (19.8%) in 2014 to 21.0 million (28.1%) in 2030, and 35.2 million (35.0%) in 2060. As a share of the total diabetes population, those aged 65 years or older accounted for 41.1% in 2014. This share would increase to 53.0% in 2030 and to 58.0% in 2060.

All race-sex groups would experience an increase in both diabetes population size and percent prevalence through 2060 (Fig. 1c and d), but the magnitude of the increase would vary. Starting with a similar percent prevalence in women (9.0%) and men (9.2%) in 2014, the rate in women would rise to 19.2%, compared to 16.6% in men, by 2060. By race, the 2014% prevalence were 12.4, 8.6, and 8.5% among blacks, whites, and people of other race, respectively. By 2060, blacks would still have the highest percent prevalence (23.3%), but prevalence in the other race group (18.8%) would exceed that in whites (16.6%). Measured in population size, however, whites with diabetes outnumbered blacks and people of other race with 16.7 million in 2014 and would continue to do so with 39.1 million in 2060.

Among all race-sex groups, black women had the highest percent prevalence in 2014 (12.5%) and would continue to have the highest rate through year 2060 (25.2%), followed by black men with 12.2% in 2014 and 21.2% in 2060. In terms of the magnitude of the increase, women of other race would have the largest relative increase in both number of cases (0.9 to 5.8 million, 6.4-fold increase) and percent prevalence (8.4 to 20.2%, 2.4-fold increase) from 2014 to 2060 (Fig. 1c, d).

Changes in diabetes incidence could have large effects on future diabetes number and percent prevalence (Tables 2 and 3). A 20% higher incidence rate than assumed in the base case would increase diabetes prevalence to 44.58 million (15.6%) in 2030 and 70.26 million (20.7%) in 2060. A 20% lower incidence would reduce diabetes prevalence to 34.7 million (12.2%) and 50.4 million (14.9%) in 2030 and 2060, respectively.

**Table 2:** Sensitivity analysis (incidence ±20%) for the projection of the future number (in millions) of US adults with diagnosed diabetes, by age group, for selected years 2014–2060

Age group (in years)					
Incidence + 20%	18–44	45–64	65–74	≥75	Total
2014	2.86	10.27	5.51	3.67	22.31
	(2.67, 3.07)	(9.79, 10.77)	(5.28, 5.75)	(3.46, 3.89)	(21.19, 23.48)
2020	4.23	13.07	8.46	5.54	31.31
	(3.87, 4.65)	(12.45, 13.75)	(8.13, 8.82)	(5.28, 5.85)	(29.72, 33.08)
2030	5.76	15.62	12.17	11.03	44.58
	(5.10, 6.54)	(14.51, 16.83)	(11.51, 12.90)	(10.43, 11.74)	(41.55, 48.02)
2040	6.16	19.1	12.82	16.75	54.83
	(5.42, 7.05)	(17.58, 20.77)	(12.01, 13.69)	(15.74, 17.91)	(50.74, 59.43)
2050	6.37	22.12	14.83	19.44	62.77
	(5.60, 7.30)	(20.33, 24.10)	(13.84, 15.88)	(18.19, 20.88)	(57.96, 68.16)
2060	6.65	23.03	18.12	22.47	70.26
	(5.83, 7.63)	(21.14, 25.11)	(16.91, 19.42)	(20.97, 24.16)	(64.85, 76.32)
Age group (in years)					
Incidence −20%	18–44	45–64	65–74	≥75	Total
2014	2.86	10.27	5.51	3.67	22.31
	(2.67, 3.07)	(9.79, 10.77)	(5.28, 5.75)	(3.46, 3.89)	(21.19, 23.48)
2020	3.45	11.12	7.55	5.09	27.22
	(3.18, 3.76)	(10.63, 11.66)	(7.27, 7.86)	(4.87, 5.35)	(25.93, 28.63)
2030	4.25	11.67	9.62	9.15	34.7
	(3.79, 4.81)	(10.88, 12.54)	(9.13, 10.17)	(8.70, 9.68)	(32.50, 37.19)
2040	4.48	13.64	9.52	12.94	40.58
	(3.96, 5.10)	(12.55, 14.85)	(8.91, 10.19)	(12.19, 13.82)	(37.60, 43.96)
2050	4.63	15.61	10.76	14.38	45.38
	(4.09, 5.29)	(14.31, 17.06)	(10.00, 11.58)	(13.43, 15.48)	(41.84, 49.41)
2060	4.84	16.24	13.07	16.26	50.4
	(4.27, 5.52)	(14.86, 17.77)	(12.12, 14.10)	(15.11, 17.59)	(46.36, 54.99)

These projections represent worst-case (high incidence) and best-case (low incidence) alternatives to the most likely base-case scenario. 95% confidence intervals are given in parentheses

**Table 3:** Sensitivity analysis (incidence ±20%) for the projection of the future percent prevalence (%) of diagnosed diabetes among US adults, by age group, for selected years 2014–2060

Age group (in years)					
Incidence + 20%	18–44	45–64	65–74	≥75	Total
2014	2.5	12.3	20.8	18.5	9.1
	(2.3, 2.6)	(11.7, 12.9)	(19.9, 21.7)	(17.4, 19.6)	(8.6, 9.5)
2020	3.5	15.5	25.5	23.7	12
	(3.2, 3.8)	(14.8, 16.4)	(24.4, 26.5)	(22.5, 25.0)	(11.4, 12.6)
2030	4.5	18.8	30.9	31.2	15.6
	(4.0, 5.1)	(17.5, 20.3)	(29.2, 32.7)	(29.5, 33.2)	(14.6, 16.8)
2040	4.7	20.8	34.8	35.8	18
	(4.2, 5.4)	(19.2, 22.6)	(32.6, 37.1)	(33.6, 38.2)	(16.6, 19.5)
2050	4.8	22.4	37.3	38.8	19.5
	(4.2, 5.5)	(20.5, 24.4)	(34.8, 39.9)	(36.3, 41.6)	(18.0, 21.1)
2060	4.8	22.8	39.1	41.5	20.7
	(4.2, 5.5)	(21.0, 24.9)	(36.5, 41.9)	(38.7, 44.6)	(19.1, 22.5)
Age group (in years)					
Incidence −20%	18–44	45–64	65–74	≥75	Total
2014	2.5	12.3	20.8	18.5	9.1
	(2.3, 2.6)	(11.7, 12.9)	(19.9, 21.7)	(17.4, 19.6)	(8.6, 9.5)
2020	2.8	13.2	22.7	21.7	10.4
	(2.6, 3.1)	(12.6, 13.9)	(21.9, 23.6)	(20.8, 22.8)	(9.9, 10.9)
2030	3.3	14.1	24.4	25.9	12.2
	(3.0, 3.8)	(13.1, 15.1)	(23.1, 25.8)	(24.6, 27.3)	(11.4, 13.0)
2040	3.5	14.9	25.8	27.6	13.3
	(3.1, 3.9)	(13.7, 16.2)	(24.2, 27.6)	(26.0, 29.5)	(12.3, 14.4)
2050	3.5	15.8	27	28.7	14.1
	(3.1, 4.0)	(14.5, 17.2)	(25.2, 29.1)	(26.8, 30.9)	(13.0, 15.3)
2060	3.5	16.1	28.2	30	14.9
	(3.1, 4.0)	(14.7, 17.6)	(26.1, 30.4)	(27.9, 32.5)	(13.7, 16.2)

These projections represent worst-case (high incidence) and best-case (low incidence) alternatives to the most likely base-case scenario. 95% confidence intervals are given in parentheses

## Discussion

Using a dynamic Markov model and improved model parameters, we have developed an updated projection of the future diabetes burden using the most recent available data. According to the current incidence during 2006 to 2014, relative mortality risk estimation, and the latest Census projection, our results indicate the number of US adults with diagnosed diabetes would continue to increase and would nearly triple from 22.3 million in 2014 to 60.6 million in 2060. The corresponding percent prevalence would double from 9% in 2014 to 18% in 2060. The magnitude of the increase would vary by age group, sex, and race. Our estimates provide essential information needed by decision-makers for future planning, preparing resources needed, and taking effective actions to combat the problem.

Diabetes already imposes large health and economic burdens on persons with the disease, their families, the national health care system, and society as a whole. Diabetes ranked seventh in the leading causes of death and contributed to 76,488 deaths in 2014. The economic cost attributed to diabetes was $245 billion in 2012 [13]. Our results indicate that these health and economic burdens of diabetes would continue to increase in the future if no actions were taken. Fortunately, type 2 diabetes, which accounts for the majority of the diabetes population, can be prevented or delayed [14]. Implementing effective prevention strategies to slow the increasing burden of type 2 diabetes is an urgent public health priority [15]. According to our analysis, if we were to reduce the diabetes incidence rate by 20%, we would reduce the number of people with diabetes by 5 million in 2030 and 10 million in 2060.

Our projection of the prevalence of diagnosed diabetes in 2030 is 39.7 million and 13.9% of the population. These estimates are higher than estimates from previous studies which ranged from 30.0 to 36.4 million and 8.6 to 10.4% [3–5, 16]. The differences between our estimates and previous studies could be due to several factors. First, our initial diabetes percent prevalence, which was derived from the most recent survey data, was higher than what previous studies predicted it would be at this time. The most recent prior projection had an initial percent prevalence of 5.5% and projected future prevalence of 6.8% in 2010 and 8.9% in 2020 [4]. However, in the real world, the actual percent prevalence was already 9.1% in 2010 [1]. A higher initial prevalence would lead to a higher projection. Second, the overall diabetes incidence estimate for 2006 to 2014 that we used for our projection was 0.8% per year, which was 10 to 30% higher than the incidence rate assumed by previous projections [3–5, 17]. This difference in the assumed incidence rate would gradually enlarge the projected diabetes prevalence for each succeeding year the model is run. Third, the estimate of relative mortality risk that we used was based on empirical data and varied across age-race-sex groups, in contrast to previous studies that used a constant value based on expert judgment. The relative mortality risk for the diabetes versus non-diabetes population that we used decreased by age (approximately 3 for age 20, 2 for age 65, and 1.2 for age 85). The lower relative mortality risk in older age groups would lead to more years of life in the large population of older people with diabetes, thereby increasing diabetes prevalence. Finally, we used the latest Census projection, which predicted a smaller future population than earlier Census projections. The 2014 Census projection predicted 400 million total population in 2050 [18], 20 million less than the 2000 Census-based projection [19]. This difference would cause a slight decrease in the projected diabetes population, but not enough to compensate for other factors that drive the increase. All of these factors combined would result in a projection that is greater in magnitude than projections from the earlier studies.

Most of the increase in future diabetes burden would be from the population 65 years and older. Starting in the late 2020s, half of the diabetes population would be in that group. Among all persons aged 65 years or older, approximately one in three in 2030 and two in five in 2060 would have diagnosed diabetes. Within the population ≥ 65 years of age, increases in the number and percent prevalence would not be distributed evenly. The increase would be more rapid in people aged 75 years or older than among those 65 to 74. The number of persons with diabetes aged 65–74 would double from 5.5 million in 2014 to 10.9 million in 2030, and triple to 15.3 million in 2060. However, the number for persons aged 75 and older would triple from 3.7 million in 2014 to 10.1 million in 2030 and increase more than fivefold to 19.5 million in 2060 (Table 1 and Fig. 1 e, f. Aging of the future US adult population [20] would play a substantial role in the rapid increase in the number of older people with diabetes. However, the increase in the age of the diabetes population would exceed the increase in age of the general population due to the higher incidence of diabetes among older people. Further, mortality reductions in people with and without diabetes would result in longer lives for those with diabetes as well as greater exposure to the risk of developing diabetes for those without the condition. All of these factors would together contribute to a steep increase in the number and prevalence of older adults with diabetes.

The increasing burden of diabetes among adults aged 65 years or older would increase heath care resources needed for this age group. The Medicare-eligible diabetes population would double in the early 2020s and quadruple in the 2050s under the current enrollment policy. The Centers for Medicare and Medicaid Services may need to consider the increased burden of diabetes when planning for future health care resources. More importantly, wide implementation of effective diabetes prevention strategies should also be considered. Lifestyle intervention is not only effective in preventing or delaying the onset of type 2 diabetes among older adults, but is also an efficient use of health care resources [21, 22].

The projected rapid increase in diabetes prevalence among blacks and people of other race compared to whites is a result of the increasing non-white population and the higher incidence of diabetes in the non-white population. Our projection suggests that racial disparities in the diabetes burden would get worse if no action were taken. We project that approximately one in four blacks would have diagnosed diabetes by year 2060. The number of women with diabetes in the other race group would increase more than six-fold from 2014 to 2060. Public policies that target prevention efforts to higher-risk groups may be needed in order to reduce the racial disparity.

Our study has limitations. First, we were not able to include youth aged 0 to 17 years in our projection due to the lack of information about this group in the NHIS. However, this would not substantially impact the total diabetes population, as diabetes is much less prevalent in the younger age group (0.25%) [23]. A previous projection of diabetes in youth estimated a 0.2 million (1.8%) prevalence of type 1 diabetes and a 0.03 million (0.27%) prevalence of type 2 diabetes in youth in 2050 [6]. Second, we did not consider undiagnosed diabetes in our projection. Adding an undiagnosed diabetes state to the model would necessitate the estimation of additional parameters. Some of these parameters, such as mortality for the undiagnosed diabetes population, the transition from undiagnosed diabetes to diagnosed diabetes, and the transition from normal glucose status to undiagnosed diabetes, would be difficult to estimate with adequate precision from available data. Further, adding an additional latent state to the model would increase the complexity of the solution and result in larger uncertainty [24]. Introducing such uncertainty would decrease the reliability of the model and could result in larger errors in the future projection. A study using data from the National Health And Nutrition Examination Survey (NHANES) that included laboratory measures estimated 8.1 million US adults (27.8% of the diabetes population) had undiagnosed diabetes in 2014 [23]. The NHANES study provided an estimate of diagnosed diabetes prevalence that was similar to our 2014 NHIS estimate for year 2014. The NHANES estimate of the undiagnosed prevalence proportion could be applied to our future projection to obtain projections of future total (diagnosed and undiagnosed) diabetes, which would be 55.0 million in 2030 and 84.0 million in 2060. Thirdly, we did not consider diabetes remission that may occur after bariatric surgery. Nevertheless, the influence would be negligible, since the number of remission cases accounts for a very small proportion of the diagnosed population and those who experience remission are at high risk of relapse [10]. Finally, similar to all projections, our projection could be biased if the key assumptions (especially regarding diabetes incidence) differ from reality. To mitigate this uncertainty, we performed a sensitivity analysis to help quantify the potential error of our projection if incidence were to change substantially.

Obesity is an important contributor to the diabetes epidemic. Previous studies considered the effect of obesity and other risk factors on the projection of diabetes burden [25] and the impact of obesity on diabetes incidence and diabetes-free life expectancy [26]. However, in our study we did not consider obesity status or other biomarkers such as blood pressure and lipid level for several reasons. First, estimates of the incidence and prevalence of obesity based on self-reported height and weight may be inaccurate. Second, the relationship between obesity and other biomarkers and diabetes is not precisely known, so diabetes incidence estimates based on these covariates might not be accurate for the purpose of our study. Third, including uncertainties such as these in a model to predict future conditions could enlarge any errors. Finally, for the purpose of predicting future prevalence, individual obesity status would be replaced by the average obesity level in each race-sex-age group, and would thus have no impact on the projection of future diabetes prevalence, the target of our study.

## Conclusion

In conclusion, using improved estimates for diabetes incidence and current prevalence, the latest census projections, and a refined mortality analysis with a dynamic Markov model, we project the number of US adults with diagnosed diabetes would nearly triple from 2014 to 2060 and over one in six adults would be diagnosed with diabetes by year 2060. The future health and economic burden imposed by diabetes on society, health care systems, and the national economy would continue to increase if no actions were taken. Wide implementation of effective prevention strategies could mitigate future increases of the diabetes burden.

## Additional file

Additional file 1: Appendix. The projection model. (DOCX 154 kb)
