# Supplementary material for: Projection of the future diabetes burden in the United States through 2060
Source: Popul Health Metr. 2018 Jun 15;16:9. doi: 10.1186/s12963-018-0166-4 (PMC6003101; doi:10.1186/s12963-018-0166-4)
Supplement: Supplementary file 1 — Appendix. The projection model. (DOCX 154 kb) [file 12963_2018_166_MOESM1_ESM.docx]

**Appendix. The projection model**

In this document, we describe the model settings and system dynamics for the projection of the future diabetes burden through 2060.

**A.1 Markov model**

The population is divided into cohorts defined by the cross-classification of race (black, white, other), sex, and age. Age is measured in single years through age 84, with persons age 85 or older grouped together. Figure 1 is a graphical depiction of model transitions from age *a - 1* to age *a* for a one-year cycle from year *t - 1* to year *t*. The same model structure applies for all ages and all race-sex groups. No Diabetes, Diabetes, and Death are the three model states. The model includes annual migration into and out of the Diabetes and No Diabetes states, based on the US Census projection. The annual transition from No Diabetes to Diabetes is determined by the analysis of incidence rates described in this paper. Transition to the Death state can occur from both the No diabetes and Diabetes states, depending on the analysis of relative mortality risks described in this paper.

Figure 1. The Markov model for disease progression for one year.

For a given race-sex cohort, the diabetes population aged *a* at year *t* has the input of the diabetes population aged *a - 1* at year t - 1 plus the newly incident diabetes population progressed from the No Diabetes to Diabetes state for age *a* in year *t*. Both the No Diabetes and Diabetes states are influenced by migration and can transit to the Death state.

**A.2 Definitions and parameters**

Note all the model settings in this section are for a given race-sex cohort.

Let *a* be the age of some population. We consider the adult population from 18 to 85 years old and take the parameter of population over 85 the same as those of 85 years old. Thus, $a\in[18,85]$.

Let *t* be the time in the model. We use integer values for *t* to represent each single year from 2012 to 2060. Let $t=0$ for the initial year 2012 and $t=48$ for 2060. So $t\in[0,48]$.

$X\left( a,t \right)$ = the number of non-diabetes population of age = *a* at year *t*.

$Y\left( a,t \right)$= the number of diabetes population of age = *a* at year *t*.

$\pi(a, t)$ = the prevalence of diabetes population of age = *a* in year *t*. Thus, $\pi\left( a, t \right)=\frac{Y(a, t)}{X\left( a, t \right)+Y(a, t)}$

$i\left( a,t \right)$ = incidence probability of diabetes among $X\left( a,t \right)$ in year *t*, estimated from NHIS.

$B\left( t \right)$ = Census projection of the number of adults turning 18 in year *t*.

$M(a, t)$ = Census projection of the number migrating at age = *a* in year *t*.

$f_{x}(t)$ = proportion of $B\left( t \right)$ without diabetes.

$f_{y}(t)$ = proportion of $B\left( t \right)$ with diabetes.

$g_{x}(a,t)$ = proportion of $M(a, t)$ without diabetes.

$g_{y}(a,t)$ = proportion of $M(a, t)$ with diabetes.

We assume the fraction of the population turning 18 years old with diabetes from 2012 to 2060 equals the diabetes prevalence for age = 18 in 2012. Thus,

$$f_{x}\left( t \right)=1-\pi(18, 0)$$

$$f_{y}\left( t \right)=\pi(18, 0)$$

We also assume the fraction of migration for people with diabetes equals the previous year’s migration for the population at the same gender, race and age. Thus,

$g_{x}\left( a,t \right)=1-\pi\left( a, t-1 \right)$*;*

$$g_{y}\left( a,t \right)=\pi\left( a, t-1 \right)$$

The relative mortality ratios between the diabetes and non-diabetes population are assumed to be time invariant in a race-sex-age cohort from 2012 to 2060.

Let

$d_{x}(a)$ = raw non-diabetes mortality rate of age = $a-1$ estimated from NHIS.

$d_{y}(a)$ = raw diabetes mortality rate of age = $a-1$ estimated from NHIS.

$\delta_{x}\left( a, t \right)$ = adjusted non-diabetes mortality rate among $X\left( a,t \right)$ in year *t*.

$\delta_{y}(a, t)$ = adjusted diabetes mortality rate among $Y\left( a,t \right)$ in year *t*.

We use the adjusted rates to match the census projection of future deaths and introduce a relative death factor $\gamma\left( a, t \right)$ to adjust them from the raw rates

$\delta_{x}\left( a, t \right)=\gamma\left( a, t \right)d_{x}(a)$ (1)

$\delta_{y}\left( a, t \right)=\gamma\left( a, t \right)d_{y}(a)$ (2)

Let

$D(a, t)$ = Census projection of the number of deaths in year *t* in the population at age = $a-1$ at the beginning of the year.

$D_{x}(a, t)$ = number of deaths in the non-diabetes population of age = $a-1$at the beginning of year *t*.

$D_{y}(a, t)$ = number of deaths in the diabetes population of age = $a-1$at the beginning of year *t*.

${D_{x}\left( a, t \right)=\delta}_{x}\left( a-1, t-1 \right)\cdot X\left( a-1, t-1 \right)$ (3)

${D_{y}\left( a, t \right)=\delta}_{y}\left( a-1, t-1 \right)\cdot Y\left( a-1, t-1 \right)$ (4)

We impose the condition $D_{x}\left( a, t \right)+D_{y}\left( a, t \right)=D(a, t)$ where $D\left( a, t \right)$equals the total number of deaths in the given race-sex-age cohort in year $t$ from the CENSUS projection. Substituting equations (1) and (2) in (3) and (4) and solving for $\gamma\left( a, t \right)$, we get,

$$\gamma(a-1,t-1)=\frac{D(a,t)}{X(a-1,t-1)d_{x}(a-1)+Y(a-1,t-1)d_{y}(a-1)}$$

$$\delta_{x}(a-1,t-1)=\frac{d_{x}(a-1)D(a,t)}{X(a-1,t-1)d_{x}(a-1)+Y(a-1,t-1)d_{y}(a-1)}$$

$$\delta_{y}(a-1,t-1)=\frac{d_{y}(a-1)D(a,t)}{X(a-1,t-1)d_{x}(a-1)+Y(a-1,t-1)d_{y}(a-1)}$$

Thus the numbers of deaths for the non-diabetes and diabetes populations are

$$D_{x}(a,t)=\frac{X(a-1,t-1)d_{x}(a-1)D(a,t)}{X(a-1,t-1)d_{x}(a-1)+Y(a-1,t-1)d_{y}(a-1)}$$

$$D_{y}(a,t)=\frac{Y(a-1,t-1)d_{x}(a-1)D(a,t)}{X(a-1,t-1)d_{x}(a-1)+Y(a-1,t-1)d_{y}(a-1)}$$

The Markov model is a discrete model in that population transitions occur annually. That is, each year is a time stage in which all events change the system states simultaneously. We assume births, migration, and incidence update the populations of the system states immediately on the current year. However, deaths update the system states in the next year. Figure 2 shows the model on a timeline with this dynamic parameter setting. Such a setting provides more precise population estimation because an individual’s death in a year still contributes to the prevalence for that year.

Figure 2. The model and parameter settings on a timeline.

Births, incidence, and migration influence the system immediately. However, deaths are reflected in the population the next year.

**A.3 System dynamics**

The transition matrix for the model ignoring migration and the starting population is given by

$$\begin{matrix} & X(a,t) & Y(a,t) & D(a,t) \\ X(a-1,t-1) & [1-\delta_{x}(a-1,t-1)]\cdot[1-i(a,t)] & [1-\delta_{x}(a-1,t-1)]\cdot i(a,t) & \delta_{x}(a-1,t-1) \\ Y(a-1,t-1) & 0 & 1-\delta_{y}(a-1,t-1) & \delta_{y}(a-1,t-1) \\ D(a-1,t-1) & 0 & 0 & 1 \end{matrix}$$

When migration and demographic changes are considered, the system can be written as a set of difference equations depending on initial conditions.

For initial conditions for the youngest age $a_{0}$,

$$X\left( a_{0},t \right)=f_{x}\left( t \right)B\left( t \right)+g_{x}\left( a_{0},t \right)M(a_{0},t)$$

$$Y\left( a_{0},t \right)=f_{y}\left( t \right)B\left( t \right)+g_{y}\left( a_{0},t \right)M(a_{0},t)$$

Given that the youngest age is 18 years, the initial population is determined by the prevalent and immigrated age 18 population. The non-diabetes and diabetes populations turning 18 at year $t$ are:

$$X\left( 18,t \right)=f_{x}\left( t \right)B\left( t \right)+g_{x}\left( 18,t \right)M\left( 18,t \right) = \left( 1-\pi\left( 18, 0 \right) \right)B\left( t \right)+\left( 1-\pi\left( 18, t-1 \right) \right)M\left( 18,t \right)$$

$$Y\left( 18,t \right)=f_{y}\left( t \right)B\left( t \right)+g_{y}\left( 18,t \right)M\left( 18,t \right) = \pi\left( 18, 0 \right)B\left( t \right)+\pi\left( 18, t-1 \right)M\left( 18,t \right)$$

For the population of age $\in[19, 84]$, the non-diabetes population is the sum of the non-diabetes population with no incidence and death, and the migration without diabetes.

$$X\left( a,t \right)=\left[ 1-\delta_{x}\left( a-1, t-1 \right) \right]\left[ 1-i\left( a,t \right) \right] X\left( a-1,t-1 \right)+g_{x}\left( a,t \right)M(a,t)$$

The diabetes population is determined by the non-diabetes and diabetes population and mortality rates in the previous year along with diabetes incidence and migration with diabetes in the current year.

$$Y\left( a,t \right)= \left[ 1-\delta_{x}\left( a-1, t-1 \right) \right] i\left( a,t \right) X\left( a-1,t-1 \right) +\left[ 1-\delta_{y}\left( a-1, t-1 \right) \right] Y\left( a-1,t-1 \right) +g_{x}\left( a,t \right)M(a,t)$$

In each race and sex cohort, the populations 85 years or older are aggregated in one age group. We need to additionally consider the population remaining in each state if not dying in the year. That is the only difference from the equations of age $\in[19, 84]$. Thus for age *a >= 85*,

$$X\left( a,t \right)=\left[ 1-\delta_{x}\left( a-1, t-1 \right) \right]\left[ 1-i\left( a,t \right) \right] X\left( a-1,t-1 \right) +\left[ 1-\delta_{x}\left( a, t-1 \right) \right]\left[ 1-i\left( a,t \right) \right] X\left( a,t-1 \right) +g_{x}\left( a,t \right)M(a,t)$$

$$Y\left( a,t \right)=\left[ 1-\delta_{x}\left( a-1, t-1 \right) \right] i\left( a,t \right) X\left( a-1,t-1 \right)+\left[ 1-\delta_{x}\left( a, t-1 \right) \right] i\left( a,t \right) X\left( a,t-1 \right)+ \left[ 1-\delta_{y}\left( a-1, t-1 \right) \right] Y\left( a-1,t-1 \right)+ \left[ 1-\delta_{y}\left( a, t-1 \right) \right] Y\left( a,t-1 \right) + g_{y}\left( a,t \right)M(a,t)$$
